# Marine Lint

**Published:** 1884-08

**Authors:** E. D. Downs

**Affiliations:** Owego, N. Y.


*Editor Independent Practitioner:*

### MARINE LINT.

*Editor Independent Practitioner:*

To those who still use cotton to fill root canals in whole or in part, or to those who use it as a vehicle to carry other materials, I would like to suggest Marine Lint instead. I have employed it to convey the chloroform solution of gutta-percha, and it may be used with any of the cements. It possesses advantages over cotton for these purposes, and seems to me to have a field of usefulness as a canal dressing. It possesses antiseptic and disinfecting properties, and will not absorb moisture. It has a long, tough fibre, and can be very easily and tightly packed.

If it is desirable to stop a root canal tightly for a time and maintain freedom of access, twist up a long rope of this and pack it closely within the canal, when you will have a tight, continuous, non-absorbent, antiseptic stopping, which may be removed by pulling upon the end left free in the cavity of decay. It may be used also as a dressing in connection with other medicaments.

Yours respectfully,

E. D. Downs, D. D. S.

Owego, N. Y., July 7, 1884.

